# Evaluating Metabolite-Based Biomarkers for Early Diagnosis of Pancreatic Cancer: A Systematic Review

**DOI:** 10.3390/metabo13070872

**Published:** 2023-07-22

**Authors:** Gloria Perazzoli, Olga M. García-Valdeavero, Mercedes Peña, Jose Prados, Consolación Melguizo, Cristina Jiménez-Luna

**Affiliations:** 1 Institute of Biopathology and Regenerative Medicine (IBIMER), Center of Biomedical Research (CIBM), University of Granada, 18100 Granada, Spain; gperazzoli@ugr.es (G.P.); omgv@correo.ugr.es (O.M.G.-V.); mpenacontreras@ugr.es (M.P.); melguizo@ugr.es (C.M.); crisjilu@ugr.es (C.J.-L.); 2 Department of Anatomy and Embryology, Faculty of Medicine, University of Granada, 18071 Granada, Spain; 3 Instituto Biosanitario de Granada (ibs.GRANADA), 18014 Granada, Spain

**Keywords:** pancreatic cancer, metabolomics, biomarkers, early diagnosis, systematic review

## Abstract

Pancreatic ductal adenocarcinoma (PDAC) is one of the deadliest cancers, with five-year survival rates around 10%. The only curative option remains complete surgical resection, but due to the delay in diagnosis, less than 20% of patients are eligible for surgery. Therefore, discovering diagnostic biomarkers for early detection is crucial for improving clinical outcomes. Metabolomics has become a powerful technology for biomarker discovery, and several metabolomic-based panels have been proposed for PDAC diagnosis, but these advances have not yet been translated into the clinic. Therefore, this review focused on summarizing metabolites identified for the early diagnosis of PDAC in the last five years. Bibliographic searches were performed in the PubMed, Scopus and WOS databases, using the terms “Biomarkers, Tumor”, “Pancreatic Neoplasms”, “Early Diagnosis”, “Metabolomics” and “Lipidome” (January 2018–March 2023), and resulted in the selection of fourteen original studies that compared PDAC patients with subjects with other pancreatic diseases. These investigations showed amino acid and lipid metabolic pathways as the most commonly altered, reflecting their potential for biomarker research. Furthermore, other relevant metabolites such as glucose and lactate were detected in the pancreas tissue and body fluids from PDAC patients. Our results suggest that the use of metabolomics remains a robust approach to improve the early diagnosis of PDAC. However, these studies showed heterogeneity with respect to the metabolomics techniques used and further studies will be needed to validate the clinical utility of these biomarkers.

## 1. Introduction

Pancreatic ductal adenocarcinoma (PDAC) is among the ten tumors with the highest mortality worldwide, approximately 500,000 in the year 2020, and this is expected to reach 800,000 by the year 2024 [1]. The most important risk factors associated with PDAC are diabetes and chronic pancreatitis [2], although there are others such as alcohol [3], cigarette smoking [4], obesity [5] and family history of PDAC [6]. The five-year survival rate is alarmingly low, only 5%, mainly attributed to late detection. This is because symptoms typically manifest in an advanced stage of the disease, resulting in a significant number of patients being diagnosed when the disease is incurable [7]. However, in patients undergoing curative surgical resection, this survival value rises to 25% [8]. Unfortunately, the vast majority of patients are diagnosed with unresectable disease (80–90%) with the serum carbohydrate antigen 19-9 (CA19-9) being the only approved marker for PDAC management. This marker is characterized by a limited usefulness due to its low specificity and sensitivity, leading to high false positive and negative rates [9,10]. This scenario emphasizes the importance of developing tools for the early detection of PDAC when the tumor is operable [11].

Metabolomics is a powerful technology that can be used to discover tumor biomarkers. One of the most-studied samples for metabolite analysis is tumor tissue, which has provided promising candidate molecules [12]. However, there is a need for further studies to find less invasive biomarkers for PDAC diagnosis, such as those detectable in peripheral blood. In recent years, different studies have been carried out to determine panels of metabolites obtained in plasma samples from PDAC patients, with promising results [13]. In addition, precursor lesions are found with increasing frequency and can be targets for early detection of this tumor type [14]. Among them, non-cystic lesion pancreatic intraepithelial neoplasia (PanIN), intraductal cystic papillary mucinous neoplasms (IPMN) and mucinous cystic neoplasms (MCN) with high dysplasia stand out due to their correlation with the development of invasive carcinoma [15]. Other non-invasive samples such as urine and saliva have also been used to determine early biomarkers in PDAC. In fact, Radon et al. [16] identify three markers, LYVE-1, REG1A and TFF1, with a sensitivity of 80% and a specificity of 76.9% that can be found in urine samples from patients with PDAC. In saliva samples, Sugimoto et al. [17] identified 48 metabolites in patients with PDAC. Of these, five metabolites (intercept, phenylalanine, tryptophan, ethanolamine and carnitine) obtained an area under the receiver operating characteristic curves (AUC) value of 0.993. However, the results were not conclusive, requiring more studies to validate their activity as early detection markers.

Finally, most studies investigate biomarkers using samples from healthy subjects as controls. However, one of the main problems in the study of biomarkers in PDAC is that they may be elevated in other non-malignant pancreatic pathologies. An example is the expression of CA19-9, which is expressed in up to 40% of patients with chronic pancreatitis [18]. However, it is important to highlight the relevance of studies that compare patients with PDAC with those with benign pancreatic diseases, especially those that increase the risk of developing this type of tumor. Mahajan et al. [19] showed the utility of an optimized and validated metabolic signature based on lipids to discriminate PDAC from chronic pancreatitis with a sensitivity of 77.3% and a specificity of 89.6%, with an overall precision of 82.4%.

Therefore, the aim of this systematic review is to discuss the most recent literature on metabolite biomarkers identified for the early diagnosis of PDAC and their potential clinical applicability in this pathology. Given the importance of finding highly sensitive and specific biomarkers, this review focuses only on studies that include comparisons between PDAC patients and those with other pancreatic diseases.

## 2. Materials and Methods

### 2.1. Research Question

This systematic review focuses on compiling all studies from the last five years that propose metabolite panels for the early diagnosis of PDAC by comparing samples from patients with this pathology with those with premalignant or non-malignant pancreatic lesions. This systematic review focuses on the last five years, continuing the review by Long et al. [20] that covers up till the year 2017. This systematic review was conducted according to the Preferred Reporting Items for Systematic Reviews and Meta-Analyses (PRISMA) 2020 statement [21]. The literature published more than five years ago was considered outdated and not included in the analysis. The registration of this review with the International Prospective Register of Systematic Reviews (PROSPERO) was requested, identification number CRD42023434856.

### 2.2. Inclusion Criteria

This systematic review included studies proposing a differential metabolite panel in patients with PDAC, including comparisons with patients with premalignant or non-malignant pancreatic lesions such as chronic pancreatitis or diabetes mellitus. For inclusion, articles were also required to be published in English between January 2018 and March 2023 and to have an accessible full text.

### 2.3. Exclusion Criteria

Articles in which the type of cancer analyzed was different from PDAC or in which only healthy individuals were used as controls were excluded. Also excluded from the analysis were studies with insufficient information on the data provided and revision articles such as systematic reviews, reviews, meta-analyses, books or editorials.

### 2.4. Data Sources

The literature search was performed in the following electronic databases: Pubmed, SCOPUS and Web of Science. The search strategy was based on Medical Subject Headings (MeSH) criteria using “Biomarkers, Tumor”, “Pancreatic Neoplasms” and “Early Diagnosis” descriptors and synonyms, resulting in the following search equation in the Pubmed database: ((Biomarkers, Tumor[MeSH Terms]) OR (“Biomarkers, Tumor”[Title/Abstract]) OR (“Biomarker*”[Title/Abstract]) OR (“marker*”[Title/Abstract])) AND ((Pancreatic Neoplasms [MeSH Terms]) OR (“Pancreatic Neoplasm*”[Title/Abstract]) OR (“Pancreas Neoplasm*”[Title/Abstract]) OR (“Pancreatic ductal adenocarcinoma”[Title/Abstract]) OR (((“Pancreas”[Title/Abstract]) OR (“Pancreatic”[Title/Abstract])) AND ((“Cancer*”[Title/Abstract]) OR (“Tumor*”[Title/Abstract]) OR (“Tumour*”[Title/Abstract]) OR (“Neoplasm*”[Title/Abstract]) OR (“Malignancy”[Title/Abstract]) OR (“adenocarcinoma”[Title/Abstract]) OR (“adenoma”[Title/Abstract]) OR (“carcinoma”[Title/Abstract])))) AND ((Early Diagnosis[MeSH Terms]) OR (“Early Diagnosis”[Title/Abstract]) OR (“Screening”[Title/Abstract]) OR (“Early Detection”[Title/Abstract]) OR (“Premature Diagnosis”[Title/Abstract]) OR (“Premature Detection”[Title/Abstract])) AND ((“Metabolite*”[Title/Abstract]) OR (“Metabolomic*”[Title/Abstract]) OR (“Metabolome”[Title/Abstract]) OR (“Metabonomic*”[Title/Abstract]) OR (“lipidome”[Title/Abstract]) OR (“Lipidomic*”[Title/Abstract])). Syntactic modifications were made to this search equation to make it compatible with the other databases mentioned above. The search was limited by filters for articles published from 1 January 2018 to 17 March 2023.

### 2.5. Study Selection

Two independent investigators (C.J.L. and G.P.) reviewed the articles obtained after the database searches. Duplicates were eliminated and articles meeting inclusion/exclusion criteria were selected by first reading the title and abstract and subsequently reading the full article. The two authors performed the selection of the studies separately, compared their results and made a joint decision.

### 2.6. Data Extraction

After the articles were selected, the data were extracted independently by the authors (G.P. and C.J.L.) according to the Cohen kappa statistical test for agreements (more than 0.8) [22]. Two other authors (O.M.G.V. and M.P.) reviewed the articles if disagreements between C.J.L. and G.P. could not be resolved by consensus. The data were extracted by G.P., C.J.L., O.M.G.V. and M.P and classified in tables according to the characteristics of the patient cohorts included in the studies (country; sample type; data set; study groups; number of subjects; cancer stage; age mean; male/female ratio) and according to the results of the differential metabolite expression analysis (list of metabolites and diagnostic values: AUC; sensibility; specificity; accuracy). Missing data were indicated by ‘not reported (NR)’. The data were sorted according to the type of sample used, except for those studies in which multiple types of samples were analyzed, which were grouped according to the sample in which the differential metabolites were found.

## 3. Results

**Figure 1:**
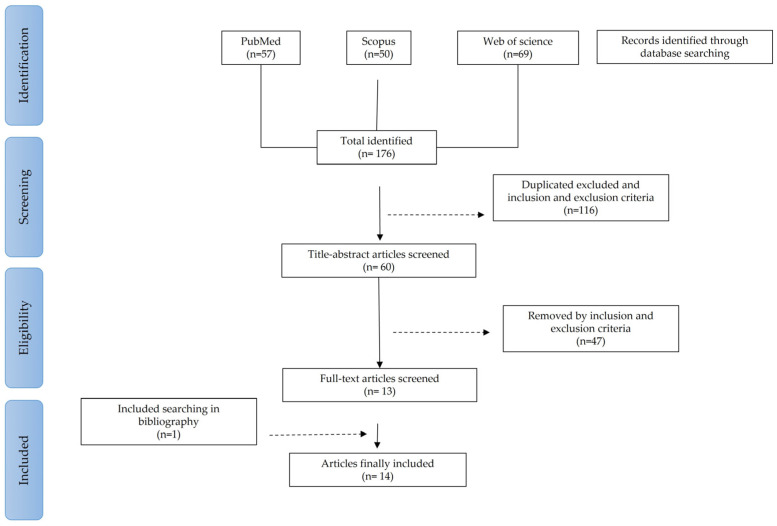
Flow diagram of articles included in this systematic review.

Figure 1 represents the flow diagram included in this systematic review. We started a total of 176 articles selected from three databases, Pubmed (*n* = 57), Scopus (*n* = 50) and Web of Science (*n* = 69). After eliminating duplicate articles and reviewing the inclusion and exclusion criteria by title and abstract reading, 60 articles were selected. Of these, 47 articles were eliminated after a review of the inclusion and exclusion criteria by full-text reading. Thirteen articles resulted from the full-text screening, to which one article was added through bibliographic search. Finally, 14 articles were included in this systematic review.

**Figure 2:**
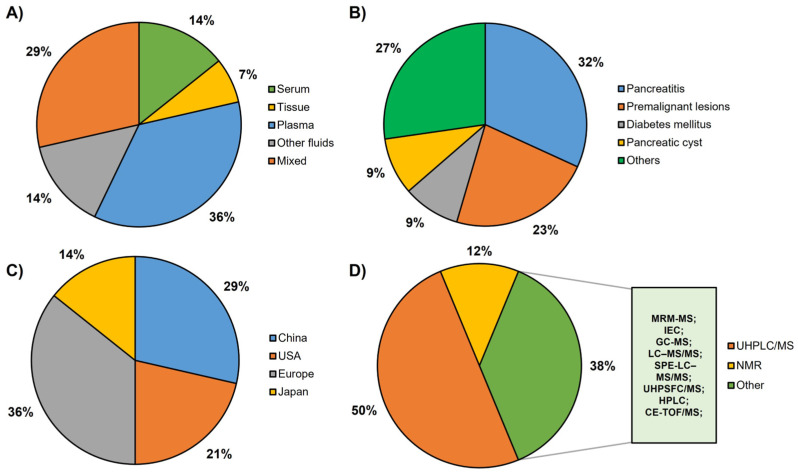
Graphical representation of the main characteristics of the studies included in this systematic review: (**A**) type of sample tested: serum, tissue, plasma, other fluids and articles with various samples. (**B**) Different types of controls included in the reviewed articles: pancreatitis, premalignant lesions, diabetes mellitus, pancreatic cyst and others. (**C**) Provenance of the cohorts: China, USA, Europe, Japan. (**D**) Techniques used for metabolomic analysis: UHPLC/MS (ultrahigh-performance liquid chromatography/mass spectrometry), including UHPLC/MS, UHPLC/QTOF-MS (UHPLC/quadrupole time-of-flight—MS) and UHPLC/ESI-QTOF-MS (UHPLC/electrospray ionization QTOF-MS); NMR (nuclear magnetic resonance), including 1H NMR (one-dimensional proton NMR) and HR-MAS NMR (high-resolution magic angle spinning NMR) and other techniques including MRM-MS (multiple reaction monitoring—MS), IEC (ion-exchange chromatography), GC-MS (gas chromatography-MS), LC–MS/MS (liquid chromatography-MS/MS), SPE-LC–MS/MS (solid-phase extraction—LC-MS/MS), UHPSFC/MS (ultrahigh-performance supercritical fluid chromatography/MS), HPLC (high-performance liquid chromatography), CE-TOF/MS (CE, capillary electrophoresis).

After conducting a comprehensive systematic review, a total of 14 articles were identified that met the predetermined inclusion and exclusion criteria. These selected articles investigated various potential metabolite-based biomarkers for PDAC by analyzing different sample sources. Out of the 14 articles, 2 utilized serum samples, 1 employed tissue samples, 5 utilized plasma samples and 2 explored other fluid samples (Figure 2A). Furthermore, four articles combined multiple sample sources to enhance their findings. Additionally, 6 of the 14 articles used validation sets to confirm the reliability of their obtained results. A very relevant aspect was the determination of the control groups used to compare the metabolomic biomarkers found in PDAC. In fact, the use of chronic pancreatitis (seven articles) and premalignant lesions (five articles), including at least one of their subtypes (IPMN, MCN and SCN), was remarkable. In addition, other pancreatic pathologies were represented, such as cystadenoma (one article), pancreatic neuroendocrine tumor (one article), diabetes mellitus (two articles), pancreatic cyst (two articles) and a mix of other types of pancreatic cancer (one article). Furthermore, two other pathologies were included as controls: liver cirrhosis (one article) and colorectal cancer (one article) (Figure 2B). Most of the studies were from European countries (five articles) and China (four articles). Furthermore, three studies with cohorts from the USA and two studies from Japan were included (Figure 2C). Based on the type of metabolic analysis carried out to obtain the different metabolites (Figure 2D), it can be seen that 50% of the articles used UHPLC/MS, with NMR being the second most used technique.

The obtained results are presented in two tables. Table 1 shows differences in terms of the origin of the cohorts studied. The average ages for the distinct types of patients analyzed were as follows: patients with PDAC, 66.4 (±4.6); pancreatitis, 55.5 (±6.8), premalignant lesions (IPMN, MCN and SCN), 61.7 (±9.5) and, finally, healthy subjects, 61 (±6.1) years old. Relevant information regarding the male/female ratio was found in the different study pathologies to be 1.2 in PDAC patients, 4 in pancreatitis, 0.82 in premalignant lesions and 1.11 in healthy individuals. Table 2 shows the most important results, highlighting the metabolites that were discovered, tested or validated. Based on this, the data of sensitivity, specificity and precision, as well as the AUC, for the different combinations of metabolites are presented.

## 4. Discussion

### 4.1. Metabolites in Tissue

Since metabolism is a hallmark of cancer, many researchers have focused their efforts on identifying metabolite-based biomarkers in different types of samples. PDAC is being extensively tested to determine the most effective types of samples and most reliable biomarkers. In this context, tumor tissue is a very rich source for the search of biomarkers, so many researchers have investigated this type of sample to discover metabolites with diagnostic value. Recently, Zhao et al. [12] conducted one of the most extensive studies using nontargeted metabolomics analysis based UHPLC-Q-TOF/MS in pancreatic tissue (PDAC and nontumor) and serum samples. First, they analyzed 105 tissue samples derived from 32 early PDAC, 19 late PDAC, 14 benign pancreatic neoplasms and 40 paired normal pancreatic tissues. Subsequently, they built a training set (*n* = 164) including serum samples from 80 PDAC patients (36 early and 44 late stages), 36 benign pancreatic neoplasms patients and 48 healthy subjects. In this study, 14 candidate biomarkers (amino acids and fatty acids) were significantly altered in both serum and tissue samples. A validation study with an independent cohort (*n* = 76) provided a panel to discriminate PDAC from non-PDAC including proline, creatine and palmitic acid. The diagnostic performance of the panel showed an AUC value to discriminate PDAC from healthy individuals of 0.854 and PDAC from benign pancreatic neoplasms of 0.865. Similarly, in the validation cohort, the AUC values were 0.830 and 0.852 for the two comparisons, respectively. Furthermore, the authors demonstrated that the combination of the metabolite panel and CA19-9 increased the diagnostic yield to discriminate early PDAC from benign conditions compared with the panel or CA19-9 individually (AUC = 0.909, 0.852, 0.757, respectively). In addition, the biomarker panel was also able to discriminate CA19-9-negative PDAC patients (*n* = 20, 25% of PDAC cases) from healthy controls, providing a sensitivity of 75.4% and specificity of 70.1% (AUC = 0.851). In the same line, Unger et al. [24] enrolled 19 early PDAC patients (I and II stages), 15 patients with benign conditions (9 paired normal pancreas, 5 pancreatitis and 1 benign pancreatic cyst), 20 patients diagnosed with high-risk premalignant lesions and 28 invasive colon cancer patients. These authors developed a six-metabolite panel (5-hydroxytryptophan, lysophosphatidylethanolamine (lysoPE) (18:2), phosphatidylcholine (PC) (16:0/16:0), PC (18:0/22:4), phosphatidylethanolamine (PE) (17:0) and sphingomyelin (SM) (d18:1/16:0)) that accurately discriminated early PDAC from benign cases (AUC = 0.95), reporting an 85% specificity and 90% sensitivity. This panel was not able to discriminate high-risk conditions from the benign group (AUC 0.46) and failed to classify colon cancer samples, suggesting PDAC specificity. Unfortunately, the validation studies carried out in plasma samples did not show reproductible abundance results. Recently, a study analyzing pancreatic tissue from humans (15 PDAC and 13 benign pancreatic lesions) and Sprague Dawley rats was performed using HR-MAS NMR [23]. Higher levels of lactate and ethanol and lower concentrations of methylene of lipid (L-CH2), myo-inositol, phosphocholine and glycerophosphocholine were detected in PDAC patients in relation to the control group. Interestingly, glycerophosphocholine, phosphocholine and myo-inositol showed lower concentrations in PDAC (humans and rats) than controls and, conversely, methanol showed higher levels. The authors did not determine the discriminatory ability of these metabolites and did not perform validation studies.

The higher lactate levels reported may derive from the well-known Warburg effect, an adaptive process through which tumor cells increase glucose uptake and its conversion to lactate [36]. In fact, lactate has been suggested as a prognostic biomarker in cancer, as high levels have been correlated with poor survival [37]. The Warburg effect also rewrites amino acid and lipid metabolisms [38], which could explain the dysregulation of proline or phospholipids such as PC and PE observed in PDAC patients. Furthermore, the dysregulation of palmitic acid levels found in the reviewed studies is in line with the association between elevated concentrations of this metabolite and increased metastatic capacity [39], suggesting a perturbed fatty acid metabolism in the context of PDAC.

### 4.2. Metabolites in Serum

The clinical implementation of the discovered biomarkers requires that they can be detectable in easily accessible and minimally invasive biological sources, so peripheral blood is one of the most desirable fluids for this purpose due to its reproducibility in clinical routine. Serum metabolome and intestinal microbiota have been related in patients with PDAC (*n* = 72) who were divided into two groups according to tumor resectability (36 PDAC stages I and II and 36 PDAC stages III and IV) [25]. Serum samples analyzed using UHPLC-Q-TOF/MS showed differential levels of some amino acids, lipids, fatty acids and carnitine derivatives between both groups of patients. Particularly, oleic acid, linoleic acid, palmitic acid, linoelaidyl carnitine, 2-octenedioic acid, 3R, 7R-1,3,7-octanetriol, lysoPE (P-16:0/0:0) and 3-hydroxyanthranilic acid individually yielded an excellent diagnostic value (AUC > 0.9). In addition, the abundance of palmitic acid, oleic acid, linoelaidyl carnitine and 2-octenedioic acid was positively correlated with certain intestinal microorganisms (*g_Anaerostipes, g_Alistipes, s_indistinctus, s_catus and s_formicigenerans*) but negatively with others (*g_Cloacibacterium, s_reuteri and s_hathewayi*). Further studies will be needed to corroborate these results. Recently, Wolrab et al. [26] conducted a wide study to determine the serum lipidomic profile in PDAC patients. It included three experimental phases focused on discovery, qualification and verification, each of them with training and validation cohorts. In Phase I and II, the authors investigated the differences between healthy controls, PDAC and pancreatitis patients by using different methods for the analysis (UHPSFC/MS, shotgun MS and MALDI-MS) and then comparing the results between three laboratories worldwide. Their results showed a different lipidomic profile between healthy controls and PDAC patients, independent of the tumor stage. The discriminatory capacity was comparable across the different methods used. Equally, the results in Phase I and Phase II showed similar patterns between the concentrations of the main dysregulated lipids. Finally, in Phase III, they investigated the applicability of serum lipidomic analysis for distinguishing PDAC and control samples. The chosen method was UHPSFC/MS, and 830 samples were interrogated in the training set, including PDAC and chronic pancreatitis patients and healthy individuals. Their results showed that the lipid species SM (41:1), SM (42:1), ceramide (Cer) (41:1), Cer (42:1), SM (39:1), lysoPC (18:2) and PC (O-36:3) were the most relevant to discriminate PDAC patients and healthy controls. Interestingly, they also observed better diagnostic performance to distinguish both groups when the lipids were combined with CA19-9 (AUC = 0.989) compared to the metabolites or CA19-9 alone (AUC = 0. 983 and 0.854, respectively). Furthermore, they found that the concentration of SM (41:1) and Cer (41:1) was downregulated in PDAC, in contrast to healthy controls and pancreatitis samples, in which they were similar, suggesting that lipidomic analysis could be a good approach for discriminating PDAC from chronic pancreatitis.

The results reported in serum-based studies suggest an alteration in lipid metabolism related to PDAC, since these are metabolites commonly obtained as potential biomarkers for this pathology when compared to different groups (benign pancreatic disease and healthy conditions). In fact, it has been demonstrated that lipids play a key role in the progression and dissemination of tumors [40]. The synthesis of fatty acids is enhanced in various types of cancer and is one of the main metabolic adaptations of tumor cells. These fatty acids serve as combustible for tumor cells and important processes such as energy production or membrane biogenesis [41]. Fatty acids and other lipid-related molecules have shown alterations in PDAC patients [42]. Due to their importance in tumorigenesis and taking into account the previous studies discussed, lipids could be a promising target for biomarker discovery.

### 4.3. Metabolites in Plasma

Plasma, a sample substance that offers advantages in terms of minimal invasiveness and routine collection in clinical settings [43], was present in eight of the fourteen studies of the review. PDAC patients were compared with different pancreatic diseases such as precancerous pancreatic cysts, chronic pancreatitis, pancreatic neuroendocrine tumors or diabetes mellitus. Considering that pancreatic cysts have a prevalence of 49.1%, it is important to develop strategies to differentiate benign pancreatic cysts from premalignant cysts, which may lead to PDAC [44]. Therefore, one of the studies compared plasma samples from PDAC patients and those with precursor pancreatic lesions such as IPMNs. In this context, Morguell et al. [28] used metabolomics approaches to validate previous markers and identify novel candidates of disease progression in plasma and cyst fluid from patients diagnosed with PDAC (*n* = 10), IPMNs, either low-grade dysplasia (*n* = 20) or high-grade dysplasia (*n* = 10) and benign serous cystic neoplasm (SCN) (*n* = 5). Using UHPLC/MS, they identified novel markers of IPMN status and disease progression, including amino acids, carbohydrates, conjugated bile acids, free and carnitine-conjugated fatty acids, purine oxidation products and trimethylamine oxide. They showed that levels of the potentially bacterial metabolites, trimethylamine oxide (AUC = 0.82) and taurochenodeoxycholate (AUC = 0.73), correlated with cyst bacterial enrichment. The microbiome appears to be closely linked to cancer-associated inflammation, making bacterial metabolites promising biomarkers for detecting PDAC [45].

In order to find biomarkers for the early detection of PDAC, some studies also used samples derived from patients with chronic pancreatitis. Mayerle et al. [33] conducted a comprehensive study consisting of both exploratory and identification phases comparing plasma and serum from PDAC patients with samples obtained from chronic pancreatitis, cirrhosis patients and healthy donors. A validation study using plasma from 79 PDAC, 80 chronic pancreatitis and 80 patients with non-pancreatic disease was also conducted to evaluate the discriminative power of the metabolic signature. Among 477 metabolites identified using GC-MS and LC-MS/MS, a biomarker panel of 9 metabolites and CA19-9 showed excellent ability to discriminate between PDAC (all stages) and chronic pancreatitis during the second phase (AUC = 0.96) and was successfully validated in the third one (AUC = 0.94). This discriminatory potential also showed excellent results in terms of distinguishing patients with resectable PDAC (stages I and II) from chronic pancreatitis in both the identification (AUC =0.99) and validation (AUC =0.93) phases. This signature included proline, SM (d18:2,C17:0), PC (C18:0,C22:6), isocitrate, sphinganine-1-phosphate (d18:0), histidine, pyruvate, Cer (d18:1,C24:0) and SM (d17:1,C18:0). An alteration in the metabolism of sphingolipids has been previously described in patients with PDAC [46,47]. Therefore, given the excellent diagnostic value obtained in this ambitious study and the clinically relevant controls included in the analyses, the implementation of this biomarker panel could improve the detection of PDAC in clinical practice. Tumas et al. [32] focused on searching for differences in plasma amino acid concentrations between PDAC (*n* = 50) and chronic pancreatitis (*n* = 7) or other pancreatic cancers (*n* = 18). Ion-exchange chromatography showed the differential expression of several amino acids between PDAC and patients with other cancers and between PDAC and a combined group including patients with other cancers or chronic pancreatitis. There was also a significant inverse correlation between PDAC stage and plasma histidine levels. Dysregulated amino acid expression could result from the increased demand to support the high growth and proliferation rate of pancreatic tumor cells. In addition, it has been reported that glutamine and arginine can promote cell proliferation and the growth of tumors [48].

On the other hand, another very extensive study based on the discovery, training and validation phases was performed for the early detection of PDAC by using plasma samples [30]. For this purpose, the discovery assay comparing patients with PDAC, chronic pancreatitis and benign pancreatic cysts and healthy individuals resulted in a five-metabolite panel that was also checked in a training (PDAC and healthy groups). A validation study was then performed in an independent cohort of 39 PDAC patients and 82 healthy controls. The results showed that this five-metabolite panel (acetylspermidine, diacetylspermine, an indole derivative and two lysoPCs) efficiently discriminated PDAC from healthy subjects (AUC = 0.903 and 0.892 in the training and validation sets, respectively) and exceeded the predictive ability of CA19-9 alone (AUC = 0.859 and 0.800 in the training and validation sets, respectively). Remarkably, when the panel was combined with a previously validated protein-based biomarker panel (CA19-9, LRG1 and TIMP1), the diagnostic performance was improved in both the training and validation sets (AUC = 0.972 and 0.924, respectively). Although this panel had an encouraging diagnostic yield, it should be noted that patients with chronic pancreatitis and benign pancreatic cysts were only included as controls in the discovery phase, but not in the final stages of training and validation. In addition, Moore et al. [31] performed UHPLC/MS analysis on samples derived from patients with PDAC (localized, locally advanced, metastatic), pancreatic neuroendocrine tumor or IPMN to assess whether metabolites could differentiate PDAC stages. Although four of them (lysine, propionyl-carnitine, C5-acylcarnitine and dodecanedioic acid) showed high correlation to PDAC, none distinguished malignancy progression. Furthermore, each disease group was associated with a group of metabolites: elevated amino acids were associated with IPMN, high levels of uric acid and methionine with pancreatic neuroendocrine tumor, locally advanced PDAC was associated with high fatty acids and polyamines and metastatic PDAC with elevated tricarboxylic acid (TCA) cycle. Local PDAC did not show overexpression of specific metabolite groups. Increased fatty acid synthesis is one of the most important aberrations in cancer cell metabolism, as it is widely recognized that fatty acids are essential for carcinogenesis and tumor cell survival [49]. Similarly, polyamine metabolism is also deregulated in several types of tumors, including PDAC [50,51].

Diabetes mellitus is another pancreatic pathology with increased risk for PDAC [52]. Therefore, some researchers analyzed this group of patients for biomarker discovery. In particular, in this review, two of the studies carried out with plasma samples compared both diseases [27,29]. First, Xu et al. [29] compared the plasma metabolic profiles of PDAC patients (*n* = 26) with diabetes mellitus (*n* = 27) and healthy volunteers using UPLC-HRMS. The results showed high expression of lysoPC (22:6 and 20:3) and 1,2,4-nonadecanetriol and low lysoPC (16:0) in the PDAC group versus the diabetes group. In fact, a panel including lysoPC (22:6), catelaidic acid, cerebronic acid, nonadecanetriol and asparaginyl-histidine discriminated PDAC from healthy controls (AUC = 0.974; 89% of sensitivity and 91% of specificity). However, the panel used to distinguish PDAC from diabetes (lysoPC (16:0, 16:1, 22:6 and 20:3)) did not provide any remarkable results (AUC = 0.723). Second, Michálková et al. [27] investigated the association of PDAC (*n* = 43) with type 2 diabetes mellitus (T2DM) (59 recent-onset and 34 long-term) through metabolomic analysis of blood plasma using 1H NMR. Seven healthy controls were also included in the study. A panel of eight metabolites (increased levels of 3-hydroxybutyrate, mannose and glutamate and decreased levels of creatine, alanine, valine, proline and lysine) was proposed and successfully tested to discriminate PDAC from patients with T2DM and from healthy controls. They also suggested that PDAC significantly alters the metabolic pathways of tryptophan and propionate, as the levels of these two metabolites were shown to be elevated in PDAC but not in T2DM patients in comparison to healthy controls. In addition, a predictive model was developed and tested in patients with recent-onset T2DM. As a result, six of these patients were classified as PDAC, and an adequate correlation with the prediction was observed as one of the patients eventually developed PDAC and three of them were later diagnosed with chronic pancreatitis. According to these results, 3-hydroxybutyric acid is implicated in promoting PDAC cell growth and progression by fueling the TCA cycle [53], while glutamate has been reported to increase the risk of precancerous pancreatic lesions becoming malignant [54].

### 4.4. Metabolites in Other Fluids

Among the other fluids used to obtain metabolites for early diagnosis in PDAC, urine, saliva and cystic fluid were found. There are few studies in this regard, although we can find some, such as the work of Ikeura et al. [34], who conducted a test for two metabolites in urine, uroporphyrin and coproporphyrin. These authors carried out a prospective study in a Japanese population of 67 patients with PDAC, comparing the results obtained for these two metabolites in 11 patients with pancreatitis and 9 healthy controls. They collected urine samples from patients and controls before and after the administration of 5-aminolevulinic acid. This causes uroporphyrin levels to increase in patients with PDAC. This metabolite may be considered as a novel biomarker for the detection of PDAC. Furthermore, both can be combined with CA19-9, increasing its sensitivity from 74.6% to 89.6%. By using saliva samples, Asai et al. [35] carried out a study in a cohort of 53 patients, 14 with chronic pancreatitis and 39 with PDAC (6 patients in stage III and 33 in stage IV), and 26 controls. In this study, they used saliva samples to validate a set of four metabolites, alanine, N1-acetylspermidine, 2-oxobutyrate and 2-hydroxybutyrate, which provided an AUC of 0.887 in the discrimination between PDAC patients (all stages) and chronic pancreatitis. Cystic fluid was used by Morguel et al. [28] in a cohort involving 15 PDAC cases, 37 patients with IPMN (8 of high and 29 of low grade) and 5 patients with SCN. From the cystic fluid, these authors validated a total of 161 metabolites, of which the most prominent were acyl-carnitines (4, 4-OH, 2, 5), choline, succinate, fumarate and malate. The acyl-carnitine group was highlighted as the best discriminator between PDAC or high-grade and non-cancerous cysts (SCN and low-grade IPMN). Among these metabolites, acyl-C4 was the one with the highest AUC, with a value of 0.83. However, the succinate presented the highest sensitivity, with a value of 90%. Regarding specificity, all the compounds presented values of 80% except acyl-C2 and succinate, which showed values of 70%. Despite the fact that the use of these fluids for the early detection of PDAC is rare, there are more and more studies that develop or validate a set of metabolites for them, since fluids such as urine or saliva are obtained easily without being invasive for the patient.

### 4.5. Metabolic Pathways

**Figure 3:**
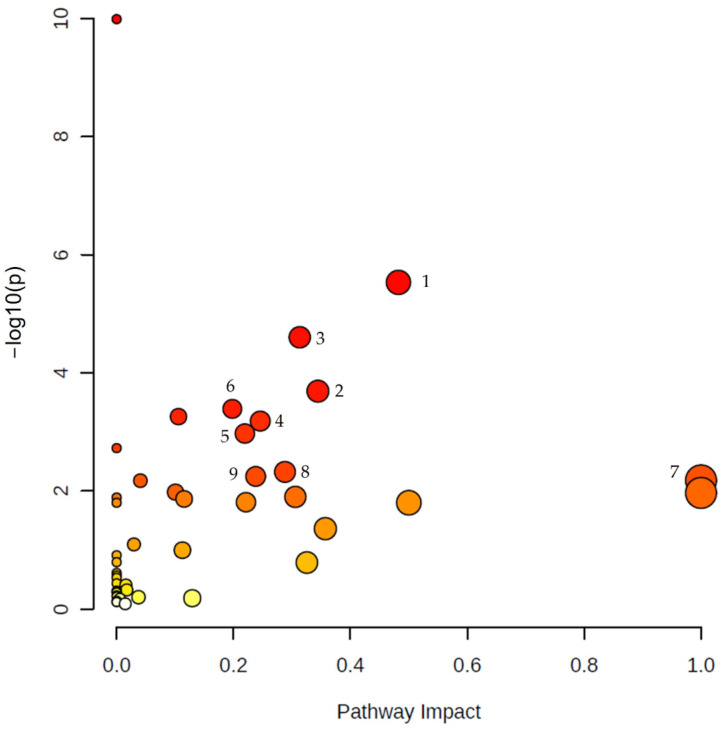
Summary of pathway analysis using Metaboanalyst. The map was obtained through online analysis (http://www.metaboanalyst.ca/ (accessed on 9 June 2023)). The significant metabolic pathways (raw *p* value < 0.05, FDR < 0.05, impact > 0.2) have been marked with numbers: (1) arginine biosynthesis, (2) arginine and proline metabolism, (3) alanine, aspartate and glutamate metabolism, (4) glycine, serine and threonine metabolism, (5) glycerophospholipid metabolism, (6) citrate cycle (TCA cycle), (7) phenylalanine, tyrosine and tryptophan biosynthesis, (8) sphingolipid metabolism, (9) pyruvate metabolism. Color gradient: yellow (less significant) to red (most significant).

MetaboAnalyst was used to analyze the altered metabolic pathways in PDAC according to the most relevant metabolites found in this systematic review. The results of the pathway analysis are presented graphically in Figure 3. The enrichment of certain metabolites in a pathway was calculated based on raw *p*-value  <  0.05 and also considering the adjusted *p*-value of the false discovery rate (FDR) < 0.05, due to the large number of pathways tested. These data are collected in Table S1. Furthermore, a pathway impact value derived from topology analysis equal to or greater than 0.2 was considered significant. A total of nine pathways were detected related to the metabolites significantly associated with PDAC, as shown in Table 2.

#### 4.5.1. Amino Acid Metabolism

Interestingly, five of the significantly perturbed metabolic pathways were related to amino acid metabolism, including (i) arginine biosynthesis, (ii) arginine and proline metabolism, (iii) alanine, aspartate and glutamate metabolism, (iv) glycine, serine and threonine metabolism and (v) phenylalanine, tyrosine and tryptophan biosynthesis. Amino acids play an important role as substrates for maintaining the energetical requirements of cancer cells. The reprogramming of amino acid metabolism is one of the mechanisms of cancer to adapt to nutrient-poor situations in the tumor microenvironment (TME) [55].

Two of the pathways that we found to be significantly altered in PDAC are related to arginine: arginine biosynthesis and arginine and proline metabolism. The alteration of arginine metabolism in PDAC has been previously described [56]. Arginine is a non-essential or semi-essential amino acid involved in several biological functions such as cell proliferation, survival and protein synthesis, and it also acts as a precursor for the synthesis of molecules involved in tumorigenesis and tumor progression in PDAC, such as nitric oxide and polyamines, among others [57,58]. Arginine biosynthesis upregulation has been reported in pancreatic tumors [59]. Arginine metabolism facilitates PDAC progression, and its depletion has been associated with the suppression of cell invasion, migration and proliferation and the promotion of autophagy and apoptosis. [56,60]. Indeed, PDAC with low argininosuccinate synthetase 1 expression, an enzyme involved in de novo arginine synthesis, may be sensitive to arginine deprivation therapy, such as arginine deiminase, making it a potential therapeutic strategy [61].

On the other hand, arginase overexpression, key in arginine metabolism, has been associated with obesity, which is known to predispose to PDAC [62]. In addition, ornithine produced by arginase serves as a precursor for proline synthesis, which supports collagen production [61]. This proline-rich collagen favors the PDAC cell metabolism, promoting tumor cell survival [63].

One of the most significantly altered pathways is alanine, aspartate and glutamate metabolism, which are non-essential amino acids related to different pathways implied in PDAC metabolism. Alanine can be converted to pyruvate, which can be oxidized in the TCA cycle or used to synthesize glucose through gluconeogenesis. Sousa et al. [64] demonstrated that stroma-associated pancreatic stellate cells secreted alanine and other non-essential amino acids that served as fuel for the TME of PDAC. In this line, Parker et al. [65] described how PDAC cells increased the expression of alanine transporters, while cells lacking transporters showed impaired metabolism, leading to decreased tumor growth.

With regard to glutamate and aspartate, both participate in transamination reactions where an amino group is transferred to a keto acid to form new amino acids. The resulting keto acids (pyruvate, oxalacetate) serve as intermediates of the TCA cycle. Meanwhile, new amino acids such as aspartate, alanine and glutamate are also produced.

The influence of glutamate in glucose metabolism has also been described. Li et al. [66] showed that glutamate produced by nerve cells enhances glycolysis in PDAC cells, promoting perineural invasion. Moreover, glutamine metabolism, which acts as a substrate for the generation of glutamate, has been described as one of the main metabolic pathways that fuels PDAC cells, together with glucose metabolism [48]. In fact, PDAC cells are especially sensitive to glutamine deprivation in the absence of asparagine [67]. These results provide evidence for the relevance of the glutamate/glutamine pathways in the context of PDAC. Aspartate is one of the main metabolites obtained from mitochondrial glutamine oxidation, and it is transported to the cytosol for nucleotide and protein biosynthesis [68]. Anglin et al. [69] demonstrated that aspartate plays an important role in the glutamine metabolism reprogramming observed in PDAC by using knockdown models both in vitro and in vivo.

Glycine, serine and threonine metabolism has also been shown to be enriched in our analysis. Serine and glycine are two highly related non-essential amino acids obtained by tumor cells through their extracellular uptake and/or intracellular synthesis, which includes de novo serine synthesis and the reversible interconversion of serine into glycine [70,71]. Thus, in a nutrient-starved environment, tumor cells are able to increase the availability of these amino acids for the construction of macromolecules, protection against oxidative stress and promotion of immunosuppression, therefore strongly supporting oncogenesis [70]. In fact, this de novo serine and glycine synthesis pathway has been observed to be upregulated in patients with non-small cell lung cancer [72]. Closely related to glycine and serine is the metabolism of threonine. Glycine can also be generated from threonine by threonine dehydrogenase. In vitro studies on mouse embryonic stem cells indicate that threonine deprivation promotes cell death by drastically reducing histone methylation [73]. Threonine is also related to the pyruvate pathway, and it is produced through the cleavage of threonine into glycine, which is converted to pyruvate by serine hydroxymethyltransferase. The pyruvate route is an important pathway in PDAC (as described below) since this amino acid can be obtained through different non-specific metabolic routes [74].

One of the most-affected metabolic routes in PDAC patients was phenylalanine, tyrosine and tryptophan biosynthesis, which showed the highest impact index in our analysis (Table S1). These aromatic amino acids are important precursors to biological compounds necessary for the proper functioning of an organism, and their alteration can lead to the development of various diseases, including cancer [75]. These three metabolites and intermediaries of this pathway have been found to be altered in some investigations included in this study, suggesting their use as PDAC biomarkers. Phenylalanine, tyrosine and tryptophan are precursors of molecules with potential for malignancy, such as phenolic and indolic compounds [75,76]. In particular, tryptophan has been reported as the metabolite most frequently altered in several cancers [77], and it has been related to immunosuppression by inhibiting antitumor immune cell proliferation and favoring T cell apoptosis, due to which it is being investigated as a target for cancer immunotherapy [78]. Furthermore, tryptophan metabolism involves the enzyme indoleamine 2,3-dioxygenase 1 (IDO1), which is a prognostic marker for PDAC that impacts on the maturation of dendritic cells [79]. Phenylalanine metabolism has also been related to the modulation of immune responses by affecting the proliferation and activation of T cells [76]. The connection between this metabolic pathway and the antitumor immune response, together with the fact that metabolic disturbances are an early event in cancer development, suggests the feasibility of investigating this pathway to identify PDAC biomarkers. In fact, altered levels of aromatic amino acids in the gastric juice of patients with early gastric cancer have been reported [80]. Similarly, these three amino acids were shown to be dysregulated in plasma samples derived from patients with esophageal cancer [81].

#### 4.5.2. Carbohydrate Metabolism

Carbohydrate metabolism has also been shown to be disturbed in our pathway analyses, revealing the role of the TCA cycle and pyruvate metabolism in PDAC. This metabolic reprogramming is driven both extrinsically to the cell through interactions with stromal cells and intrinsically by mutated KRAS signaling, which is very common in this type of cancer [82]. Pyruvate metabolism is key in the cellular metabolic network, being a link between the cytosolic and mitochondrial metabolisms. The cytosolic pyruvate can either remain in the cytosol to be converted into lactate or it can be transported into the mitochondria [83]. Specifically, lactate was one of the metabolites included in this systematic review, and it was reported to be increased in PDAC patients [23]. This is in line with the excessive production of lactate derived from glucose uptake by tumor cells observed in cancer patients, leading to the acidification of TME and, consequently, to immunosuppression, metastasis and tumor angiogenesis [83,84]. Moreover, under conditions of limited glucose, lactate can be incorporated into the TCA cycle and be a metabolic fuel for tumor cells [84]. Other metabolites also involved in carbohydrate metabolism and reported as PDAC biomarkers in this review are succinate, fumarate and malate, among others. Hassan et al. [85] studied the serum metabolic profile of patients with breast cancer and observed an altered carbohydrate metabolism in obese patients. On the other hand, other authors demonstrated that the carbohydrate metabolism was related to the outcome of chemotherapy in PDAC patients, showing upregulated levels of intermediates of the TCA cycle and glycolysis, such as pyruvate, which were associated with non-response after gemcitabine-based chemotherapy [86]. These data suggest a key role for carbohydrate metabolism in the development and poor prognosis of PDAC, making this metabolic process a target in the search for biomarkers.

#### 4.5.3. Lipid Metabolism

Pathway analyses also showed altered lipid metabolism, highlighting the importance of glycerophospholipid metabolism and sphingolipid metabolism in PDAC, while linoleic acid metabolism emerged among the pathways with the highest, although not significant, impact (Table S1). Glycerophospholipid metabolism is currently understood as relevant to cancer development and progression [87]. Among the lipids belonging to this pathway, phosphatidylserine, PC and PE were suggested as biomarkers. Kaoutari et al. [88] found a significant relationship between the expression of glycerophospholipids in PDAC and the prediction of patient survival and resistance to gemcitabine, closely related to the multi-drug resistance phenotype. Other authors such as Wang et al. [89] analyzed the sphingolipid pathway in addition to the glycerophospholipid pathway, concluding that both were deregulated in PDAC. Alterations in this lipid metabolism were also related to the initiation and development of this tumor type. It has been seen that sphingolipid metabolism is highly related to the different metabolic subtypes of PDAC. In their study, Mahajan et al. [90] showed that the metabolism of sphingolipids can divide PDAC patients into three subtypes according to the altered metabolic pathways. By analyzing the metabolic pathways of sphingolipids, glycolipids and phospholipids, it was observed that the first subtype presented a mixed phenotype of metabolic pathways, while phenotype 2 showed an enrichment of and subtype 3 a reduction in these routes, with the most commonly altered pathway being the sphingolipid metabolism. These metabolites are related to the biosynthesis of unsaturated fatty acids, apoptosis and autophagy. In relation to apoptosis, tumor cells can modify their metabolism to increase cell survival and avoid cell death. This can be achieved through several routes, such as increasing the levels of complex sphingolipids that have anti-apoptotic functions and, therefore, drug resistance, or accumulating ceramide or sphingosine-1-phosphate to increase cell survival, among others [91].

On the other hand, long-chain polyunsaturated fatty acids such as linoleic acid may promote the progression of PDAC as they are related to the energy generation needed by tumor cells to grow. This fatty acid has been shown to be significantly decreased in patients with unresectable PDAC compared to patients with resectable PDAC [25]. Although no significant FDR results (FDR > 0.05) were obtained related to the pathway of this metabolite in the analysis carried out in this systematic review, it is a metabolic route with the highest impact (Table S1). This may be due to the fact that there is evidence of the involvement of fatty acids in the survival of the tumor cells and in the development of metastases [49].

Despite the fact that a number of new biomarkers for the early diagnosis of PDAC have been reported in recent years, there is no firm molecular list to be implemented in clinical routine. Our results support those of Long et al. [20] in their previous review on metabolite-based biomarkers in PDAC, with amino acid metabolism being the most enriched, highlighting the (i) alanine, aspartate and glutamate, (ii) glycine, serine and threonine and (iii) arginine and proline pathways. In addition, after analyzing the results of the present systematic review, we suggest that the phenylalanine, tyrosine and tryptophan biosynthesis pathway, also related to amino acid metabolism, is altered in PDAC patients. Moreover, based on our results, we propose that lipid metabolism should be considered as a target in the search for biomarkers since it is significantly altered in PDAC patients. In addition, unregulated glycolysis is a hallmark of cancer, and several metabolites of this pathway have been studied as potential biomarkers, such as glucose and lactate in PDAC tissue and body fluids, although metabolomics is constantly evolving.

One of the most outstanding limitations that we can find after carrying out this review is the lack of information about the patients in some articles, such as data related to the tumor phase. Another limitation is related to the techniques used in the selected articles, highlighting the need to standardize methodologies used by different authors to allow translational comparisons of the results obtained. Furthermore, it is necessary to underline the importance of the metabolite validation stage, as it consolidates the findings obtained during the initial discovery phase. Although this step is present in most of the articles, it is not available for all metabolites, requiring more studies to validate the relevance of the data obtained. In addition, the diagnostic value of the metabolites studied has not been indicated in all the articles analyzed, which is very important to develop an accurate panel for the early detection of PDAC. On the other hand, the lack of information regarding all the metabolites identified in the reviewed articles could result in additional metabolic pathways involved in PDAC not reported in this review, since our pathway analysis was performed with the most relevant metabolites listed by the authors.

## 5. Conclusions

PDAC is a highly lethal disease with a low survival rate due to its late diagnosis. Current diagnostic markers, such as CA19-9, have limited sensitivity and specificity, highlighting the need for new biomarkers for early detection. Metabolomics has emerged as a promising approach to identify potential biomarkers for this disease. Our systematic review shows promising metabolite panels with diagnostic capacity to be applied in clinical oncology to the diagnosis of PDAC, demonstrating the potential of this technology to identify new diagnostic biomarkers for this tumor type. Some of the studies discussed in this review used samples from patients with pre-malignant lesions at high risk for PDAC. However, to our knowledge, no metabolomics-based fingerprints have yet been found to classify this group with acceptable accuracy. Therefore, studies that include this group of patients for the biomarker discovery phase are necessary in order to anticipate the development of PDAC. Promising candidates for future metabolite studies based on the results of the present systematic review should focus on the key components of two main perturbed metabolic processes: amino acid and lipid metabolisms. In this sense, it would be interesting to study tryptophan and glutamine as promising candidate molecules from amino acid metabolism and pyruvate-related metabolites such glycine, alanine and threonine. Regarding the metabolism of lipids, SM and PC, which have been detected in tissue, plasma and serum samples, could represent reliable biomarkers for early PDAC detection. In addition, the association of metabolite panels with CA19-9 has been shown to provide greater diagnostic performance. Nevertheless, it is also important to investigate potential biomarkers in CA19-9-negative patients, as they constitute about 25% of PDAC cases according to previous observations. Although metabolites are one interesting approach to improve the prognosis of PDAC patients through early detection, further studies will be necessary for their validation as useful pancreatic cancer biomarkers.

## Figures and Tables

**Table 1:** Principal demographic characteristics of the studies included.

Study	Cohort Country	Samples	Data Set	Study Groups	Cancer Stage (n)	Age Mean	M/F
[12]	China	Tissue	Discovery	PDAC (51)	I (13), II (19), III (12), IV (7)	60.9	29/22
				Precursor lesions (14): IPMN (3), MCN (4), SCN (7)	-	55.35	8/6
				Paired nontumor pancreatic tissue (40)	-	63.35	23/17
		Serum	Training	PDAC (80)	I (17), II (19), III (23), IV (21)	61.2	48/32
				Precursor lesions (36): IPMN (15), MCN (9), SCN (12)	-	58.05	23/13
				Healthy subjects (48)	-	60.35	31/17
		Serum	Validation	PDAC (22)	I (8), II (14)	66.91	11/11
				Precursor lesions (27): IPMN (6), MCN (7), SCN (14)	-	59.15	15/12
				Healthy subjects (27)	-	58.37	17/10
[23]	China	Tissue	Discovery	PDAC (15)	-	NR	NR
				Benign pancreatic disease (13): Cystadenoma and congenital cyst		NR	NR
[24]	United States	Tissue and plasma	Training (tissue), validation (plasma)	PDAC (19)	IA, IB, IIA	61.57	13/6
				Benign pancreatic disease (15): pancreatitis and pancreatic cystic neoplasms		50.73	6/9
				Precursor lesions (20): IPMN (14), MCN (3), others (3)	-	57.9	7/13
				Colorectal adenocarcinoma (28)	-	60	NR
[25]	China	Serum	Whole data	Unresectable PDAC (36)	III (9), IV (27)	63	NR
				Resectable PDAC (36)	I(30), II (6)	59	NR
[26]	Czech Republic	Serum	Training (Phase III)	PDAC (430)	T1, T2, T3, T4	NR	219/211
				Healthy subjects (246)	-	NR	122/124
				Pancreatitis (22)	-	NR	13/9
			Validation (Phase III)	PDAC (116)	T1, T2, T3, T4	NR	56/60
				Healthy subjects (16)	-	NR	6/10
[27]	Czech Republic	Plasma	Whole data	PDAC (43)	-	67	26/17
				Healthy subjects (29)	-	63	10/19
				T2DM (34)	-	68	18/16
				RODM (59)	-	65	27/32
[28]	Sweden	Plasma	Whole data	PDAC (10)	-	76	6/4
				IPMN LGD (20)	-	68	10/10
				IPMN HGD (10)	-	74	3/7
				SCNs (5)	-	47	0/5
		Cyst fluid	Whole data	PDAC (15)	-	71	7/8
				IPMN LGD (29)	-	70	12/17
				IPMN HGD (8)	-	76	3/5
				SCNs (5)	-	47	0/5
[29]	China	Plasma	Whole data	PDAC (26)	-	64.74	14/12
				DM (27)	-	56.93	14/13
				Healthy subjects (23)	-	60.22	12/11
[30]	United States	Plasma	Training (Cohort 1)	PDAC (20)	IB (2); IIA (1); IIB (7); IV (10)	70.4	10/10
				Healthy subjects (10)	-	60.2	4/6
				Healthy subjects (60)	-	62.0	30/30
				Chronic pancreatitis (10)	-	61.6	6/4
			Training (Cohort 2)	PDAC (9)	IA (2); IIA (2); IIB (2); IV (4)	73.1	3/6
				IPMNs (34), MNC (11), SCN (6)	-	62.2	19/32
			Validation	PDAC (39)	IA (6); IB (10); resectable No TNM data (22)	62.0	21/18
				Healthy subjects (82)	-	62.8	43/39
[31]	United States	Plasma	Whole data	IPMN (10)	-	66	6/4
				Localized PDAC (10)	-	63	4/6
				Locally advanced PDAC with nodal disease (10)	-	70	6/4
				Unresectable metastatic (10)	-	64	6/4
				PNET (10)	-	64	8/2
[32]	Lithuania	Plasma	Whole data	PDAC (50)	IA (4); IB (4); IIA (5); IIB (21); III (16)	65.5	28/22
				Other pancreatic cancers (18)	-	63.5	10/8
				Chronic pancreatitis (7)	-	58	4/3
[33]	Germany	Plasma	Exploratory study	PDAC (34)	IB (1); IIA (4); IIB (8); III (11) and IV (10)	64	15/19
				Chronic pancreatitis (43)	-	50	36/7
				Liver cirrhosis (20)	-	56	15/5
				Healthy subjects and non-pancreatic disease control blood donors (104)	-	53	49/55
		Serum and plasma	Training	PDAC (158): serum (80) and plasma (78)	IA (2); IB (3); IIA (18); IIB (59); III (22); IV (54)	70	102/56
				Chronic pancreatitis (159): serum (79) and plasma (80)	-	50	136/23
				Liver cirrhosis (80): serum (80)	-	61	60/20
				Non-pancreatic disease control blood donors (77): serum (77)	-	55	51/26
		Plasma	Validation	PDAC (79)	IA (1); IIA (11); IIB (28); III (26); IV (13)	69	37/42
				Chronic pancreatitis (80)	-	51	62/18
				Non-pancreatic disease preoperative patients (80)	-	68	42/38
[34]	Japan	Urine	Validation	PDAC (67)	-	72	48/19
				Pancreatitis (11)	-	67	8/2
				Healthy subjects (9)	-	69	9/0
[35]	Japan	Saliva	Development	PDAC (39)	III (6), IVa (12), IVb (21)	66.1	21/18
				Chronic pancreatitis (14)	-	51.1	11/3
				Healthy subjects (26)	-	50.8	13/13

DM, diabetes mellitus; F, female; HGD, high-grade dysplasia; IPMN, intraductal papillary mucinous neoplasm; LGD, low-grade dysplasia; M, male; MCN, mucinous cystic neoplasm; NR, not reported; PDAC, pancreatic ductal adenocarcinoma; PNET, pancreatic neuroendocrine tumor; RODM, recent-onset diabetes mellitus; SCN, serous cystic neoplasm; T2DM, type 2 diabetes mellitus.

**Table 2:** The performances of the principal metabolites obtained from included studies.

Study	Sample	Metabolomics Approach	Data Set	More Relevant Comparison	Results (Metabolites)	AUC (CI-CI)	Sens. (%)	Spec. (%)	Accuracy (%)
[23]	Tissue	HR-MAS NMR	Discovery	PDAC vs. benign pancreatic disease	Lactate and ethanol > in PDAC. Methylene of lipid (L-CH2), myo-inositol, phosphocholine and glycerophosphocholine < in PDAC	NR	NR	NR	NR
[24]	Tissue	UHPLC/ESI-Q-TOF-MS	Discovery	Early PDAC vs. benign pancreatic disease	6-metabolite panel: 5-hydroxytryptophan, LysoPE (18:2), PC (16:0/16:0), PC (18:0/22:4), PE (17:0) and SM (d18:1/16:0)	0.95 (0.78–1)	90	85	NR
				High-risk PDAC vs. benign pancreatic disease	6-metabolite panel: 5-hydroxytryptophan, LysoPE (18:2), PC (16:0/16:0), PC (18:0/22:4), PE (17:0) and SM (d18:1/16:0)	0.46 (0.21–0.73)	NR	NR	NR
					12 metabolites: 1-Indanol (with 12 different *m*/*z*)	0.836 (0.57–0.98)	NR	NR	NR
	Plasma	MRM-MS	Validation	Early PDAC vs. benign pancreatic disease	6-metabolite panel: 5-hydroxytryptophan, LysoPE (18:2), PC (16:0/16:0), PC (18:0/22:4), PE (17:0) and SM (d18:1/16:0)	Failure in the analysis due to discordant metabolite abundance results with respect to tissue.	NR	NR	NR
[27]	Plasma	1H NMR	Whole data	PDAC vs. healthy subjects	Increased: 3-hydroxybutyrate and mannose Decreased: creatine, ornithine, alanine, uridine, serine, histidine, carnitine, glutamine, glycine, threonine, lysine and methionine	NR	NR	NR	NR
				T2DM vs. healthy subjects	Increased: glucose Decreased: 18 metabolites (ornithine, uridine, histidine and glutamine)	NR	NR	NR	NR
				PDAC vs. T2DM	Increased: 3-hydroxybutyrate, propylene glycol, mannose, propionate, glutamate and tryptophan Decreased: creatine, alanine, valine, proline and lysine	NR	NR	NR	NR
				PDAC vs. (T2DM and healthy subjects)	Increased: 3-hydroxybutyrate, mannose and glutamate Decreased: creatine, alanine, valine, proline and lysine	NR	NR	NR	NR
[28]	Plasma	UHPLC/MS	Whole data	(HGD or PDAC) vs. (LGD or SCNs)	Bacterial metabolite trimethylamine oxide (9.12 µM)	0.82 (0.65–0.94)	80	90	NR
					Taurochenodeoxycholate (204 nM)	0.73 (0.56–0.87)	80	70	NR
[29]	Plasma	UHPLC/MS	Whole data	PDAC vs. (DM and healthy subjects)	Increased: lysoPC (22:6), lysoPC (20:3) and 1,2,4-nonadecanetriol Reduced: lysoPC (16:0)	NR	NR	NR	NR
				(PDAC and DM) vs. healthy subjects	Increased: lysoPC (20:4), deoxyadenosine, asparaginyl-histidine and vaccenyl carnitine Reduced: phytal, 2 (R)-hydroxydocosanoic acid, behenic acid, catelaidic acid, 2-hydroxyphytanic acid, phytosphingosine, cerebronic acid, docosanamide and eicosenoic acid	NR	NR	NR	NR
				PDAC vs. healthy subjects	Combination 1: lysoPC (22:6), catelaidic acid, cerebronic acid, docosanamide and asparaginyl-Histidine	0.882 (0.846–0.918)	81.6 (76.0–87.2)	87.3 (83.4–91.2)	85.2 (82.1–88.3)
					Combination 2: lysoPC (16:0), catelaidic acid, cerebronic acid, nonadecanetriol and asparaginyl-histidine	0.974 (0.958–0.991)	89.0 (84.7–93.3)	90.6 (86.1–95.1)	88.6 (86.4–90.9)
					Combination 3: lysoPC (22:6), catelaidic acid, cerebronic acid, docosanamide and asparaginyl-histidine	0.879 (0.848–0.909)	83.4 (78.1–88.7)	89.6 (86.0–93.2)	86.5 (84.3–88.8)
					Combination 4: lysoPC (16:0), lysoPC (16:1), lysoPC (22:6) and lysoPC (20:3)	0.860 (0.823–0.896)	84.8 (78.5–91.1)	83.1 (77.8–88.3)	83.7 (80.1–87.4)
					Combination 5: lysoPC (16:0), lysoPC (16:1), lysoPC (22:6) and lysoPC (20:3), catelaidic acid, cerebronic acid, docosanamide, nonadecanetriol and asparaginyl-histidine	0.919 (0.887–0.952)	89.4 (85.3–93.5)	77.3 (71.4–83.2)	84.1 (80.8–87.4)
					CA19-9	0.821 (0.765–0.874)	79.1 (74.5–82.6)	82.6 (76.5–89.4)	81.3 (77.8–83.4)
				PDAC vs. DM	Combination 1 (same as PDAC vs. healthy subjects)	0.586 (0.534–0.638)	50.3 (43.9–56.6)	62.9 (57.5–68.3)	58.6 (55.6–61.5)
					Combination 2 (same as PDAC vs. healthy subjects)	0.631 (0.580–0.682)	54.7 (49.3–60.0)	61.3 (54.4–68.3)	54.7 (52.2–57.2)
					Combination 3 (same as PDAC vs. healthy subjects)	0.569 (0.516–0.622)	46.3 (39.8–52.7)	61.2 (54.3–68.1)	54.7 (51.6–57.9)
					Combination 4 (same as PDAC vs. healthy subjects)	0.723 (0.691–0.754)	63.5 (58.9–68.1)	69.6 (64.1–75.1)	67.7 (64.1–71.3)
					Combination 5 (same as PDAC vs. healthy subjects)	0.680 (0.632–0.729)	58.9 (53.5–64.3)	67.1 (60.9–73.2)	65.9 (62.6–69.2)
[30]	Plasma	UHPLC/MS	Training	PDAC vs. healthy subjects	5-marker metabolite panel: (N1/N8)-acetylspermidine (AcSperm), DAS, lysoPC (18:0), lysoPC (20:3) and an indole-derivative	0.903 (0.818–0.989)	NR	NR	NR
					CA19-9	0.859 (0.743–0.975)	NR	NR	NR
					Protein (CA19-9, TIMP1 and LRG1)	0.948 (0.883–1.000)	NR	NR	NR
					Protein (CA19-9, TIMP1 and LRG1) + metabolite multiplex panel	0.972 (0.928–1.000)	NR	NR	NR
			Validation	PDAC vs. healthy subjects	Indole-derivative	0.726 (0.631–0.822)	23.1 (95% specificity)	11.3 (95% sensitivity)	NR
					LysoPC (18:0)	0.842 (0.764–0.920)	51.3 (95% specificity)	26.3 (95% sensitivity)	NR
					LysoPC (20:3)	0.841 (0.757–0.925)	48.7 (95% specificity)	11.3 (95% sensitivity)	NR
					AcSperm	0.755 (0.659–0.852)	33.3 (95% specificity)	27.5 (95% sensitivity)	NR
					DAS	0.801 (0.712–0.890)	51.3 (95% specificity)	27.5 (95% sensitivity)	NR
					5-marker metabolite panel	0.892 (0.828–0.956)	66.7 (95% specificity)	43.3 (95% sensitivity)	NR
					CA19-9	0.800 (0.708–0.891)	NR	NR	NR
					Protein (CA19-9, TIMP1 and LRG1)	0.863 (0.782–0.946)	NR	NR	NR
					Protein (CA19-9, TIMP1 and LRG1) + metabolite multiplex panel	0.924 (0.864–0.983)	NR	NR	NR
[31]	Plasma	UHPLC/MS	Whole data	Higher correlation with disease state than CA19-9	Four metabolites: lysine, propionyl-carnitine, C5-acylcarnitine and dodecanedioic acid	NR	NR	NR	NR
				PNET	High: uric acid, methionine	NR	NR	NR	NR
				IPMN	High: amino acid	NR	NR	NR	NR
				Locally advanced PDAC	High: fatty acid and polyamines	NR	NR	NR	NR
				Metastatic PDAC	High: TCA cycle metabolites	NR	NR	NR	NR
				Local PDAC	No predominance of specific principal components	NR	NR	NR	NR
[32]	Plasma	Ion-exchange chromatography	Whole data	PDAC vs. (OPC and chronic pancreatitis)	Ornithine, threonine, phenylalanine, glycine, arginine, histidine, glutamine, 3-methylhistidine and citruline	NR	NR	NR	NR
				PDAC vs. OPC	Ornithine, threonine, phenylalanine, lysine, valine, arginine, histidine, asparagine, glutamine, 3-methylhistidine and citruline	NR	NR	NR	NR
				Different PDAC stages	Inverse correlation between plasma histidine concentrations and PDAC stage. U-shaped curves from stage I to stage IV were observed for tyrosine, proline, glycine, arginine, serine and threonine	NR	NR	NR	NR
[33]	Plasma	GC-MS; LC–MS/MS; SPE-LC–MS/MS	Validation/test	PDAC (all stages) vs. chronic pancreatitis	Biomarker signature	0.94 (0.91–0.97)	89.9 (81–95.5)	91.3 (82.8–96.4)	90.6 (84.9–94.6)
					CA19-9	0.85	NR	NR	NR
				Resectable PDAC (stages IA-IIB) vs. chronic pancreatitis	Biomarker signature	0.93	90.0 (76.3–97.2)	91.3 (82.8–96.4)	90.8 (84.2–95.3)
					CA19-9	0.84	NR	NR	NR
				PDAC (all stages) vs. don-pancreatic controls	Biomarker signature	0.9	NR	NR	NR
					CA19-9	0.89	NR	NR	NR
				Resectable PDAC (stages IA-IIB) vs. Non-pancreatic controls	Biomarker signature	0.88	NR	NR	NR
					CA19-9	0.88	NR	NR	NR
	Plasma and serum		Training	PDAC (all stages) vs. chronic pancreatitis	Biomarker signature: CA19-9 and nine metabolites (Proline; SM (d18:2,C17:0); PC (C18:0,C22:6); isocitrate; sphinganine-1-phosphate (d18:0); histidine; pyruvate; Cer (d18:1,C24:0); SM (d17:1,C18:0))	Plasma: 0.96 (0.93–0.98) Serum: 0.88	Plasma: 94.9 (87–97)	Plasma: 85	Plasma: 90 (86–91)
					CA19-9	Plasma: 0.88 Serum: 0.8	NR	NR	NR
				Resectable PDAC (stages IA-IIB) vs. chronic pancreatitis	Biomarker signature	Plasma: 0.99 Serum: 0.81	Plasma: 98.2 (93.3–99.4)	Plasma: 85	Plasma: 91.6 (89.2–92.2)
					CA19-9	Plasma: 0.91 Serum: 0.7	NR	NR	NR
				PDAC (all stages) vs. liver cirrhosis	Biomarker signature	Serum: 0.87	NR	NR	NR
					CA19-9	Serum: 0.79	NR	NR	NR
				Resectable PDAC (stages IA-IIB) vs. liver cirrhosis	Biomarker signature	Serum: 0.79	NR	NR	NR
					CA19-9	Serum: 0.7	NR	NR	NR
				PDAC (all stages) vs. healthy subjects	Biomarker signature	Serum: 0.95	NR	NR	NR
					CA19-9	Serum: 0.88	NR	NR	NR
				Resectable PDAC (stages IA-IIB) vs. healthy subjects	Biomarker signature	Serum: 0.87	NR	NR	NR
					CA19-9	Serum: 0.79	NR	NR	NR
[12]	Serum	UHPLC/Q-TOF-MS	Training	PDAC vs. healthy subjects	Proline, creatine and palmitic acid	0.854 (0.842–0.865)	80	79.2	79.7
					Proline, creatine and palmitic acid + 19-9	0.919 (0.911–0.928)	82.5	89.6	85.2
				Early PDAC vs. healthy subjects	Proline, creatine and palmitic acid	0.880 (0.864–0.896)	88.9	79.2	83.3
					Proline, creatine and palmitic acid + CA19-9	0.900 (0.886–0.915)	86.1	85.4	85.7
				PDAC vs. precursor lesions	Proline, creatine and palmitic acid	0.865 (0.800–0.931)	76.3	86.1	NR
					CA19-9	0.806 (0.719–0.892)	75	86.1	72.2
					Proline, creatine and palmitic acid + CA19-9	0.917 (0.868–0.966)	86.3	86.1	NR
				CA19-9 negative PDAC patients vs. healthy subjects	Proline, creatine and palmitic acid	0.851 (0.840–0.863)	75.4	70.1	72
			Validation	Early PDAC vs. healthy subjects	Proline, creatine and palmitic acid	0.83 (0.792–0.866)	76.2	70.4	72.9
					Proline, creatine and palmitic acid + 19-9	0.949 (0.933–0.966)	85.7	81.5	83.3
				Early PDAC vs. precursor lesions	Proline, creatine and palmitic acid	0.852 (0.736–0.967)	86.4	77.8	NR
					CA19-9	0.757 (0.616–0.897)	77.3	74.1	73.5
					Proline, creatine and palmitic acid + CA19-9	0.909 (0.825–0.993)	81.8	88.9	NR
[25]	Serum	UHPLC/Q-TOF-MS	Whole data	Unresectable PDAC vs. resectable PDAC	Oleic acid	0.965 (0.922–0.991)	NR	NR	NR
					Linoleic acid	0.979 (0.945–0.998)			
					Palmitic acid	0.984 (0.957–1)			
					Linoelaidyl carnitine	0.965 (0.918–0.992)			
					2-Octenedioic acid	1 (1–1)			
					3R,7R-1,3,7-Octanetriol	0.984 (0.949–1)			
					LysoPE (P-16:0/0:0)	0.981 (0.947–1)			
					3-Hydroxyanthranilic acid	0.957 (0.902–0.989)			
[26]	Serum	UHPSFC/MS	Training and Validation	PDAC vs. healthy subjects	CA19-9	0.854	70.33	97.33	79.08
					Lipids	0.983	95.97	90.46	94.18
					CA19-9 + lipids	0.989	95.97	92.75	94.93
			Training	PDAC vs. healthy subjects	The lipid species with the highest relevance are SM (41:1), SM (42:1), Cer (41:1), Cer (42:1), SM (39:1), LysoPC (18:2) and PC (O-36:3)	NR	NR	NR	NR
[34]	Urine	HPLC	Validation	Normal pancreas, PDAC and pancreatitis	CA19-9	NR	74.6	83.3	76.5
					UP	NR	55.2	70	58.6
					CP	NR	41.8	85	51.7
					CA19-9 + UP	NR	83.6	57.9	77.9
					CA19-9 + CP	NR	86.6	68.4	82.6
					CA19-9 + UP + CP	NR	89.6	52.6	81.4
[35]	Saliva	CE-TOF/MS	Development	Normal pancreas, PDAC and chronic pancreatitis	Alanine, N1-acetylspermidine, 2-oxobutyrate and 2-hydroxybutyrate	0.887 (0.784–0.944)	NR	NR	NR
[28]	Cyst fluid	UHPLC/MS	Whole data	(SCN and LGD) vs. (HGD and PDAC)	Acyl-C4 (0.237 µM)	0.83 (0.69–0.93)	80	80	NR
					Acyl-C4-OH (0.0751 µM)	0.79 (0.67–0.90)	80	80	NR
					Acyl-C2 (6.94 µM)	0.78 (0.65–0.89)	80	70	NR
					Acyl-C6 (0.0374 µM)	0.77 (0.63–0.89)	80	80	NR
					Choline (7.06 µM)	0.78 (0.65–0.89)	70	80	NR
					Succinate (2.89 µM)	0.80 (0.67–0.91)	90	70	NR
					Fumarate (1.11 µM)	0.76 (0.62–0.89)	70	80	NR
					Malate (20.5 µM)	0.78 (0.64–0.90)	70	80	NR
				SCN vs. PDAC	5-Oxoproline (247 µM)	1 (1–1)			NR
					Glutamine (264 µM)	1 (0.935–1)			NR
					Ethanolamine phosphate	1 (0.935–1)	100	90	NR
					D-Glucose	0.971 (0.871–1)	100	90	NR
				HGD vs. PDAC	Indole	0.89 (0.655–1)	90	90	NR
					L-Adrenaline	0.85 (0.63–0.98)	90	70	NR
					Malate	0.78 (0.52–0.96)	70	80	NR
					S-Adenosyl-L-methionine	0.83 (0.61–0.96)	70	90	NR
					Dopamine	0.83 (0.635–0.96)	60	90	NR
					Tryptophan	0.82 (0.61–0.96)	80	70	NR

1H, one-dimensional proton; AUC, area under the curve; CE, capillary electrophoresis; Cer, ceramide; CI, confidence interval; CP, coproporphyrin; DAS, diacetylspermine; DM, diabetes mellitus; ESI, electrospray ionization; GC, gas chromatography; HGD, high-grade dysplasia; HP, high-performance; HR, high-resolution; IPMN, intraductal papillary mucinous neoplasm; LC, liquid chromatography; LGD, low-grade dysplasia; LysoPC, lysophosphatidylcholine; LysoPE, lysophosphatidylethanolamine; MAS, magic angle spinning; MCN, mucinous cystic neoplasm; MRM, multiple reaction monitoring; MS, mass spectrometry; NMR nuclear magnetic resonance; NR, not reported; OPC, other pancreatic cancers; PC, phosphatidylcholine; PDAC, pancreatic ductal adenocarcinoma; PNET, pancreatic neuroendocrine tumor; Q-TOF, quadrupole time-of-flight; RODM, recent-onset diabetes mellitus; SCN, serous cystic neoplasm; Sens, sensitivity; SM, sphingomyelin; SPE, solid-phase extraction; Spec, specificity; T2DM, type 2 diabetes mellitus; UHPLC, ultrahigh-performance liquid chromatography; UP, uroporphyrin.

## Data Availability

The data presented in this study are available in the main article and the supplementary materials.

## Supplementary Materials

The following supporting information can be downloaded at: https://www.mdpi.com/article/10.3390/metabo13070872/s1, Table S1: Result from pathway analysis.
