# Supplementary material for: Evaluating Metabolite-Based Biomarkers for Early Diagnosis of Pancreatic Cancer: A Systematic Review
Source: Metabolites. 2023 Jul 22;13(7):872. doi: 10.3390/metabo13070872 (PMC10384620; doi:10.3390/metabo13070872)
Supplement: Supplementary file 1 [file metabolites-13-00872-s001.zip › metabolites-2472050-supplementary.pdf]

SUPPLEMENTARY MATERIAL

Evaluating metabolite-based biomarkers for early diagnosis of pancreatic cancer: a systematic review

Table S1. Result from Pathway Analysis.

	Total	Expected	Hits	Raw p	-log10(p)	Holm adjust	FDR	Impact
**Aminoacyl-tRNA biosynthesis**	48	1,6413	14	1,02E-10	9,9915	8,566E-06	8,566E-06	0
**Arginine biosynthesis**	14	0,4787	6	2,93E-06	5,5338	0,0002428	0,0001229	0,48223
**Alanine, aspartate and glutamate metabolism**	28	0,9574	7	2,49E-05	4,6041	0,0020404	0,0006967	0,3133
**Arginine and proline metabolism**	38	1,2994	7	0,0002035	3,6915	0,01648	0,0042726	0,34441
**Citrate cycle (TCA cycle)**	20	0,6839	5	0,0004055	3.392	0,032441	0,0068127	0,19798
**Glyoxylate and dicarboxylate metabolism**	32	1,0942	6	0,0005486	3,2607	0,04334	0,0076805	0,10582
**Glycine, serine and threonine metabolism**	33	1,1284	6	0,000653	3,1851	0,050937	0,0078365	0,24577
**Glycerophospholipid metabolism**	36	1.231	6	0,001061	2,9743	0,081697	0,011141	0,21938
**Valine, leucine and isoleucine biosynthesis**	8	0,2736	3	0,0018738	2,7273	0,14241	0,017489	0
**Sphingolipid metabolism**	21	0,7181	4	0,0047373	2,3245	0,3553	0,039793	0,28804
**Pyruvate metabolism**	22	0,7523	4	0,0056448	2,2484	0,41771	0,043046	0,23794
**Phenylalanine, tyrosine and tryptophan biosynthesis**	4	0,1368	2	0,0065884	2,1812	0,48095	0,043046	1
**Propanoate metabolism**	23	0,7865	4	0,0066618	2,1764	0,48095	0,043046	0,04061

**Glycolysis / Gluconeogenesis**	26	0,889	4	0,010423	1.982	0,74007	0,060147	0,10065
**Linoleic acid metabolism**	5	0,171	2	0,010741	1.969	0,75184	0,060147	1
**Tyrosine metabolism**	42	1,4361	5	0,012547	1,9015	0,86576	0,063038	0,30581
**Butanoate metabolism**	15	0,5129	3	0,012843	1,8913	0,87332	0,063038	0
**Glutathione metabolism**	28	0,9574	4	0,013571	1,8674	0,90926	0,063038	0,11548
**Histidine metabolism**	16	0,5471	3	0,015429	1,8117	1	0,063038	0,22131
**Nitrogen metabolism**	6	0,2052	2	0,01576	1,8025	1	0,063038	0
**D-Glutamine and D-glutamate metabolism**	6	0,2052	2	0,01576	1,8025	1	0,063038	0,5
**Phenylalanine metabolism**	10	0,3419	2	0,043311	1,3634	1	0,16537	0,35714
**Porphyrin and chlorophyll metabolism**	30	1,0258	3	0,080023	1,0968	1	0,29226	0,02955
**Cysteine and methionine metabolism**	33	1,1284	3	0,10018	0,99921	1	0,35064	0,11254
**Biosynthesis of unsaturated fatty acids**	36	1.231	3	0,12218	0,91301	1	0,41051	0
**Synthesis and degradation of ketone bodies**	5	0,171	1	0,15986	0,79626	1	0,50496	0
**Tryptophan metabolism**	41	1,4019	3	0,16231	0,78966	1	0,50496	0,32556
**Ascorbate and aldarate metabolism**	8	0,2736	1	0,24344	0,6136	1	0,73033	0
**Ubiquinone and other terpenoid-quinone biosynthesis**	9	0,3077	1	0,26945	0,56953	1	0,78046	0
**Biotin metabolism**	10	0,3419	1	0,29457	0,53081	1	0,8248	0
**alpha-Linolenic acid metabolism**	13	0,4445	1	0,36497	0,43774	1	0,98895	0
**Pyrimidine metabolism**	39	1,3335	2	0,3884	0,41072	1	1	0,01584
**Primary bile acid biosynthesis**	46	1,5729	2	0,47222	0,32585	1	1	0,01735
**Pantothenate and CoA biosynthesis**	19	0,6497	1	0,48572	0,31362	1	1	0

**Selenocompound metabolism**	20	0,6839	1	0,50352	0,29798	1	1	0
**Ether lipid metabolism**	20	0,6839	1	0,50352	0,29798	1	1	0
**beta-Alanine metabolism**	21	0,7181	1	0,52072	0,2834	1	1	0
**Lysine degradation**	25	0,8548	1	0,58386	0,23369	1	1	0
**Galactose metabolism**	27	0,9232	1	0,61229	0,21304	1	1	0
**Phosphatidylinositol signaling system**	28	0,9574	1	0,62578	0,20358	1	1	0,03736
**Inositol phosphate metabolism**	30	1,0258	1	0,6514	0,18615	1	1	0,12939
**Purine metabolism**	65	2,2226	2	0,66223	0,17899	1	1	0,0054
**Arachidonic acid metabolism**	36	1.231	1	0,71836	0,14366	1	1	0
**Fatty acid elongation**	39	1,3335	1	0,74693	0,12672	1	1	0
**Fatty acid degradation**	39	1,3335	1	0,74693	0,12672	1	1	0
**Valine, leucine and isoleucine degradation**	40	1,3677	1	0,75581	0,12159	1	1	0
**Fatty acid biosynthesis**	47	1,6071	1	0,80994	0,091547	1	1	0,01473

The statistical p values from enrichment analysis are further adjusted for multiple testings. In particular, the Total is the total number of compounds in the pathway; the Hits is the actually matched number from the user uploaded data; the Raw p is the original p value calculated from the enrichment analysis; the Holm p is the p value adjusted by Holm-Bonferroni method; the FDR p is the p value adjusted using False Discovery Rate; the Impact is the pathway impact value calculated from pathway topology analysis.
